# Genetic Diversity and Population Structure of *Leishmania infantum* in Morocco as Revealed by Multilocus Sequence Typing (MLST) Approach

**DOI:** 10.3390/pathogens12060785

**Published:** 2023-05-31

**Authors:** Sara El Mazini, Mourad Barhoumi, Idris Mhaidi, Othmane Daoui, Mouad Ait Kbaich, Sofia El Kacem, Imane El idrissi Saik, Myriam Riyad, Khadija Bekhti, Ikram Guizani, Meryem Lemrani

**Affiliations:** 1 Laboratory of Parasitology and Vector-Borne-Diseases, Institut Pasteur du Morocco, Casablanca 20360, Morocco; 2 Laboratory of Microbial Biotechnology and Bioactive Molecules, Faculty of Sciences and Technologies, Sidi Mohammed Ben Abdellah University, Fes 30000, Morocco; 3 Molecular Epidemiology and Experimental Pathology (MEEP)/ LR16IPT04, Institut Pasteur de Tunis, Université de Tunis El Manar, B.P. 74, Tunis 1068, Tunisia; 4 Laboratory of Cellular and Molecular Pathology, Research Team on Immunopathology of Infectious and Systemic Diseases, Faculty of Medicine and Pharmacy, Hassan II University of Casablanca, Casablanca 21100, Morocco

**Keywords:** visceral leishmaniasis, cutaneous leishmaniasis, *Leishmania infantum*, *Leishmania tropica*, MLST, genetic diversity, gene flow, Morocco

## Abstract

*Leishmania infantum* is endemic in Morocco, and it causes both visceral (VL) and cutaneous leishmaniasis (CL). In this study, the multilocus sequence typing (MLST) approach was used to investigate the phylogeny and population structure of *Leishmania infantum* strains isolated from CL and VL patients and the canine reservoir in different leishmaniasis endemic foci in Morocco. For this purpose, eight loci (*pgm*, *alat*, *me*, *fh*, *g6pd*, *pgd*, *gpi* and *cytb*) were amplified in 40 samples, out of which 31 were successfully sequenced. The genetic diversity analysis detected a high degree of intraspecific genetic variability among the studied strains. The phylogenetic and the haplotype analyses showed that most of the strains from the same geographical areas clustered together. The recombination among *Leishmania infantum* strains was revealed through a splits tree analysis and the number of recombination events. Moreover, the assessment of the gene flow between *Leishmania infantum* and *Leishmania tropica* through phylogenetic analysis and haplotype diversity in two endemic foci where the two species were sympatric showed no genetic exchange between the two species.

## 1. Introduction

Globally, over 20 species of the genus *Leishmania* are causative agents of a broad spectrum of diseases called leishmaniasis, ranging from self-limiting localized cutaneous lesions to visceral leishmaniasis with a fatal outcome [1]. Leishmaniasis are endemic in 98 countries, and about 350 million people are at risk of acquiring them [2].

Visceral leishmaniasis (VL) and cutaneous leishmaniasis (CL) are endemic in Morocco. The species *Leishmania (L.) infantum* can cause both forms. The first case of VL due to zymodeme MON-1 was reported in 1920 in Tangier province [3]. VL is mainly distributed in the north of the country where the disease was originally described, with cases being reported in the center and the south [4].

The early clinical symptoms of VL include intermittent fever, malaise and shivering. The overt disease manifests by striking splenomegaly and is either accompanied or not by hepatomegaly. The hyperplasia of the reticuloendothelial system is accompanied by wasting and pallor of the mucous membranes [5,6].

As for the cutaneous form caused by *L. infantum* MON-24, El Mazini et al. (2022) reported that it was first identified in 1996 in the north in Taounate province [7]. Thereafter, many cases were notified in diverse areas throughout the country, and most of them were foci where *L. tropica* was endemic or where this species was reported, indicating a change in *Leishmania* distribution with *L. infantum* invading *L. tropica* endemic areas such as, for instance, Sefrou [8] and Taza [9].

The cutaneous lesions caused by *L. infantum* are usually represented as a small single ulcerated or lupoid lesion on the face that lastsup to three years [10,11,12]. Nevertheless, clinical polymorphism has been observed with erythematous, papular or nodular lesions [13,14].

Dogs are the main reservoir of zymodeme MON-1 [15], and zymodeme MON-24 was also isolated from a domestic dog [16]. Moreover, rodents were found to be infected by *Leishmania infantum* in endemic cutaneous leishmaniasis zones [17]. Three proven phlebotomine species of the *Larroussius* subgenus act as *Leishmania infantum* vectors: *Phlebotomus ariasi*, *Phlebotomus longicuspis* and *Phlebotomus perniciosus* [9,18,19].

Different methods to identify, classify and characterize *Leishmania* species were developed, and each one has its advantages and disadvantages. Multilocus enzyme electrophoresis (MLEE), long considered as the gold standard for species and strain typing of *Leishmania*, is based on 15 enzymatic systems analyzed by starch gel electrophoresis [20]. However, this method is quite slow, laborious, costly, necessitates large amounts of in vitro parasite culture and it has a low discriminatory power due to its inability to detect silent nucleotide substitutions [21].

For these reasons, PCR-based assays were developed and proved their usefulness for the description of species and even strain diversity [22]. Multilocus sequence typing (MLST), first proposed for bacterial molecular epidemiology investigations, has powerful phylogenetic potential and is likely to become the new gold standard method for *Leishmania* species characterization [23] due to the complex genomic composition of protozoan parasites such as species of the *Leishmania* genus. These parasites display a high nucleotide diversity among markers corresponding to housekeeping gene coding regions [24].

El Hamouchi et al. (2018) reported many changes in the eco-epidemiological profile of *L. infantum* in Morocco, such as the southward spread of the parasite and the occurrence of treatment-resistant *L. infantum* [25]. Moreover, the emergence of new cases of CL due to *L. infantum* was reported in different parts of the country where this form of leishmaniasis has never been notified [7]. We still need to better understand the dynamics of leishmaniasis due to *L. infantum* and the population structure of this species through genetic analysis. This could help us to assess the risk of spread of both CL and VL caused by *L. infantum*. To date, few studies have been conducted on the population genetics of *L. infantum* in Morocco despite the importance of this species.

In this survey, we used the MLST approach to study the genetic diversity, phylogeny and population structure of *L. infantum* strains isolated from CL and VL patients and the canine reservoir in different endemic foci in Morocco, including those where *L. infantum* was co-sympatric with *L. tropica*. To evaluate the possible gene flow between these two species, we also studied the genetic diversity of *L. tropica* strains collected in these co-endemic areas.

For this purpose, we used a scheme of seven housekeeping genes used in MLEE and as DNA markers in MLST studies [24,26,27] in addition to the kinetoplast marker cytochrome b (*Cyt b*) [28].

## 2. Materials and Methods

### 2.1. Ethics Statement

Informed consent was obtained from all adults who participated in this study. Consent for the inclusion of young children was obtained from their parents or guardians.

### 2.2. Samples

**Figure 1:**
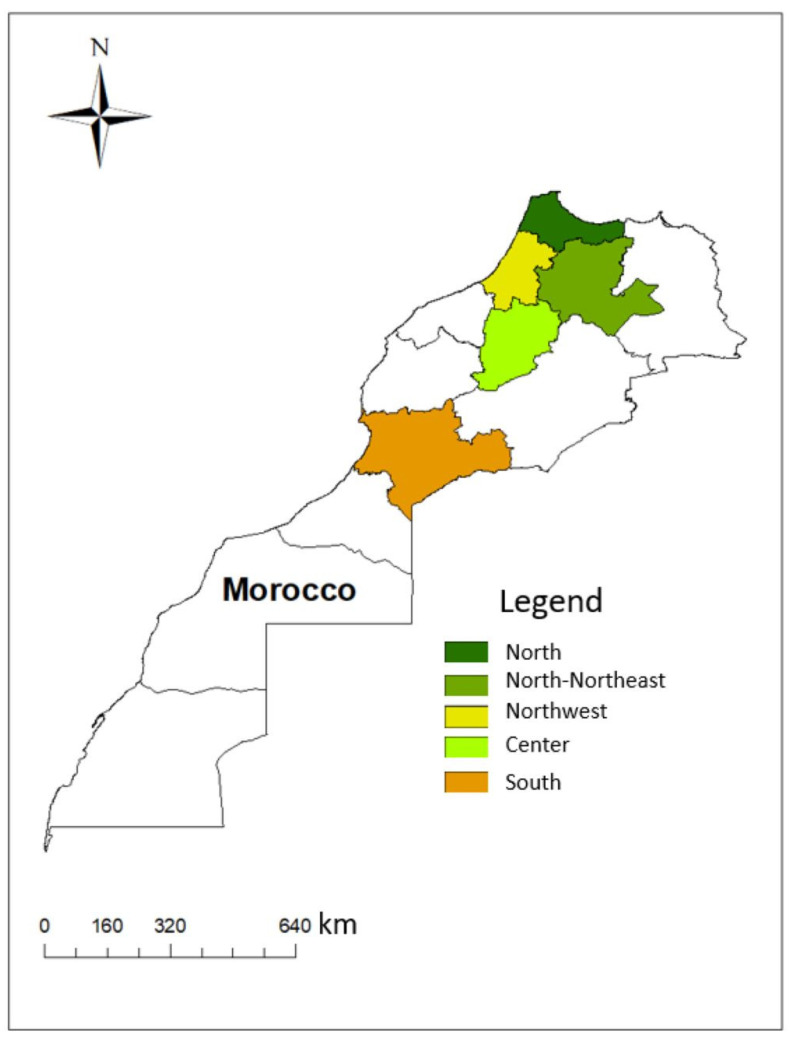
Map showing the sampling areas.

The human visceral leishmaniasis (VL) samples were collected by iliac crest puncture by a pediatrician from patients hospitalized at the university hospital in Fes that came from VL endemic regions in Morocco (Table 1, Figure 1).

The cutaneous leishmaniasis (CL) samples of *L. infantum*, including one HIV+/CL and *L. tropica,* were collected by dermal scraping from the edge of the lesions in northern Morocco (Table 1, Figure 1).

As for the canine VL samples, they were collected in the framework of the surveys we conducted in northern Morocco from lymph nodes of suspected dogs. We also used Moroccan *L. infantum* canine strains kindly provided by Pr. J.P. Dedet (the CHU Montpellier, centre national de référence des leishmanioses) (Table 1, Figure 1). All human and dog samples were microscopically examined on slide smears after fixation with absolute methanol and Giemsa staining using a ×100 immersion objective.

### 2.3. DNA Extraction and Leishmania Species Identification

For DNA extraction from *L. infantum* cultures, we used the phenol-chloroform method as described in [29]. From CL samples, the DNA was extracted from dermal scraping using an ISOLATEII Genomic DNA commercial kit (Bioline, London, UK) following the manufacturer’s instructions.

To detect and identify *Leishmania* species, the ITS1 coding region was first amplified using the primers LITSR and L5.8S following the protocol of Schonian et al. (2003) [30]. Then, the positive samples were digested by Hae III restriction enzyme for 2 h at 37 °C. RFLPs were visualized by electrophoresis on 3% agarose gel containing ethidium bromide along with the weight marker HyperLadder 100 bp (Bioline, London, UK). The profiles assigned the DNA samples to the *Leishmania* species.

### 2.4. PCR Amplification of the Eight Markers

Seven metabolic enzyme-coding genes, namely malic enzyme (*me*), fumarate hydratase (*fh*), glucose-6-phosphate dehydrogenase (*g6pdh*), glucose-6-phosphate isomerase (*gpi*), phosphogluconate dehydrogenase (*pgd*), alanine aminotransferase (*alat*), phosphoglucomutase (*pgm*) and mitochondrial cytochrome b gene (*Cyt b*), were amplified from each sample (Table 2).

The optimized PCR reactions for the amplification of these eight DNA markers were carried out in reaction volumes of 25 μL containing 1× PCR reaction buffer, 1.5 mM MgCl_2_, 1.5 U Taq DNA polymerase (Bioline, London, UK), 0.4 mM primers (Table 2), 3 μL template DNA and distilled water to reach 25 μL. The amplification reactions were conducted in an S1000 thermal cycler (Bio Rad, Hercules, CA, USA) following a cycling program for denaturation at 95 °C for 4 min, then 30 amplification cycles (94 °C for 30 s, 58 °C for 30 s and 72 °C for 90 s) and a final elongation at 72 °C for 10 min. This protocol was optimized for the amplification of the eight markers. The PCR products were visualized in 1% agarose gel containing ethidium bromide along with molecular weight marker HyperLadder 100 bp (Bioline, London, UK).

### 2.5. PCR Products Sequencing

The amplified PCR products were purified using an Ex’S-Pure™ enzymatic PCR cleanup kit (NimaGen, Nijmegen, The Netherlands). Then, using the same sets of primers, they were sequenced in both directions using a BrillantDye Terminator Cycle Sequencing Kit v.1.1 (NimaGen, Nijmegen, The Netherlands) and an ABI PRISM 3130xL DNA automated sequencer (Applied Biosystems, Waltham, MA, USA).

### 2.6. Genetic Diversity and Phylogeny Analyses

The sequences of each locus amplified were aligned and compared to entries retrieved from GenBank, inspected to check the heterozygous sites, edited and trimmed using the Chromas v.2.6.2 (Technelysium) and MEGA software v.11 [31].

We used the software Dnasp v6 [32] to calculate the number of polymorphic/heterozygous sites (S), number of haplotypes (H), haplotype diversity (Hd), nucleotide diversity (Pi), the average number of nucleotide differences (K) and the ratio of synonymous and non-synonymous substitutions (dN/dS).

To estimate population differences, the analysis of molecular variance (AMOVA) was performed using the Arlequin software v.3.5.2.2 [33] to calculate the level of genetic diversity among different populations. It indicates how much of this variance is due to differences between or within populations. We performed the AMOVA analysis among the strains based on the disease form VL, CL forms and CanL.

Along with AMOVA, we calculated the differentiation index (Fst), a common measure of the degree of genetic differentiation. This index varies from zero (no genetic differentiation) to one (the population has reached fixation for the different alleles).

Tajima’s D neutrality test [34] is based on the distribution of allele frequency of segregating nucleotide sites. Positive values indicate a bias toward intermediate-frequency alleles, while negative values indicate a bias toward rare alleles, which signifies a recent population expansion or selective sweep. Tajima’s D neutrality test was calculated for all groups.

We concatenated the sequences using the MLSTest software v.1.0.1.23 [35]. The same software was used to estimate the genotypic variation where the allele types (ATs) were calculated, and each unique sequence was given an allelic profile, based on which strains were assigned a sequence type (ST) number. The typing efficiency (TE) and discriminatory power (DP) were calculated for each locus.

We used the MLSTest software v.1.0.1.23 [35] to carry out a congruence test of all the loci with 10,000 replicates, which suggested a homogeneity of all the loci with a *p* value of 1, and to create the phylogenetic trees using the bio-neighbor joining (BIONJ) algorithm. The heterozygous sites were established as average states in order to avoid possible phylogenetic signal misinterpretation during the analysis, and 1000 replications were used to evaluate the significance of the nodes.

We visualized and edited the resulting trees with TreeGraph2 [36]. We used the population genetics software PopART v.1.7 to build the haplotype network using the median joining network algorithm and to visualize it [37].

### 2.7. Recombination Evaluation

The recombination amongst *L. infantum* isolates was evaluated through split decomposition analysis using the neighbor-net method implemented in SplitsTree4 v4.18.3 software [38]. The splits tree analysis was performed using alignments of the concatenated sequences of all seven loci of *L. infantum* isolates. The software Dnasp v6 was used to calculate the estimate of recombination (R) per gene and the minimum number of recombination events [39,40].

### 2.8. Temporal Variation Analysis

We assessed the temporal variation in the different sequence types (STs) across the years of sampling using Excel 2013.

### 2.9. Analysis of the Genetic Exchange between L. infantum and L. tropica

To assess the possibility of gene flow between the two endemic *Leishmania* species, *L. infantum* and *L. tropica,* in Morocco, a set of 12 *L. tropica* sequences were retrieved from Genbank (https://www.ncbi.nlm.nih.gov/genbank/) accessed on 12 August 2022 (Table 3); the same samples were amplified for the markers alanine aminotransferase (*alat*) and phosphoglucomutase (*pgm*). In addition, two *L. tropica* strains from northern Morocco were amplified for the same loci (Table 1, Figure 1).

## 3. Results

### 3.1. Leishmania infantum Identification and Markers Amplification

Based on the ITS1-PCR-RFLP analysis, all the 40 samples were identified as *L. infantum*. These samples were amplified and sequenced for eight markers (Table 2). The *cytb* marker was excluded due to the bad quality of sequences. Thirty one sequences out of the forty samples were successfully sequenced for the seven loci (supplementary data Figure S1); the sequences were submitted to GenBank and are accessible using the following accession numbers: OQ627483 to OQ627513 (for *alat*), OQ627514 to OQ627544 (for *fh*), OQ627547 to OQ627577 (for *g6pd*), OQ627580 to OQ627610 (for *gpi*), OQ627613 to OQ627643 (for *me*), OQ627646 to OQ627676 (for *pgd*) and OQ627679 to OQ627709 (for *pgm*). The samples were divided into three groups: cutaneous leishmaniasis (CL), visceral leishmaniasis (VL) and canine leishmaniasis (CanL) (Table 1).

### 3.2. Leishmania infantum Genetic Diversity Analyses

To assess the diversity of the studied strains, we estimated the genetic diversity indices of all of the *L. infantum* strains together and for visceral, cutaneous and canine leishmaniasis for the *L. infantum* strains separately.

#### 3.2.1. Genetic Diversity of All *L. infantum* Strains

The analyzed fragments comprised between 412 bp (*alat*) and 594 (*g6pd*) for the 31 *L. infantum* strains. The concatenated sequences of 3368 bp length recorded 82 polymorphic/heterozygous sites, 18 haplotypes and a high haplotype diversity of 0.892, and the average number of nucleotide differences was 5.694.

The analysis of all of the *L. infantum* strains in each locus showed that the marker *pgd* had the highest number of polymorphic/heterozygous sites with 25 sites, followed by *g6pd* with 16 and *gpi* with 12. In addition, the highest number of haplotypes were recorded at these loci (Table 4). The haplotype diversity, nucleotide diversity and the average number of nucleotide differences were also calculated (Table 4); all the loci had a dN/dS value < 1, indicating negative or purifying selection, except for *fh,* which had a dN/dS = 1, indicating a neutral selection.

#### 3.2.2. Genetic Diversity of Canine and Human Groups

Sixty-eight and seventeen polymorphic/heterozygous sites were recorded in the canine and human groups, respectively; the latter group had thirteen haplotypes, while the canine group had nine haplotypes. As for the haplotype diversity and the average number of nucleotide differences, they were higher in the canine strains with values of 0.895 and 12.006, respectively. The genetic diversity indices indicated *pgd* as the most polymorphic locus in both groups with twenty-two and four polymorphic/heterozygous sites in the canine and the human groups, respectively, while the marker *fh* was the least polymorphic in the canine group and showed zero segregating sites in the human group (Table 5).

The human group contained VL and CL strains, and therefore we analyzed these two sub-groups separately.

#### 3.2.3. Genetic Diversity of the Human VL and CL Sub-Groups

The concatenated sequences of the human CL sub-group showed twelve polymorphic/heterozygous sites, while the human VL sub-group had nine; seven haplotypes were recorded in both sub-groups. The haplotype diversity and the average number of nucleotide difference were higher in the CL strains with values of 0.864 and 3.167, respectively. Three markers, *pgd*, *me* and *gpi* had two as the highest number of polymorphic/heterozygous sites in the VL sub-group, while the loci *gpi* and *me* had three as the highest number of polymorphic/heterozygous sites in the CL sub-group; the marker *fh* showed zero polymorphic/heterozygous sites in both groups along with *pgm* in the CL sub-group (Table 6).

The canine samples (*n* = 9) had a higher number of polymorphic/heterozygous sites in comparison with the human group (*n* = 22); however, the latter had more haplotypes. Inside the human group, the CL sub-group (*n* = 6) had a higher number of segregating sites than the VL sub-group (*n* = 16). Nevertheless, both groups had the same number of haplotypes.

### 3.3. Demographic Analysis and Genetic Structure

Tajima’s D test rejected neutrality with negative values in all groups, suggesting a recent population expansion or selective sweep. However, inside the human group, the human VL strains had a positive value of 0.18667, indicating a bias toward intermediate-frequency alleles. Tajima’s D was negative in the CL strains with a value of −0.85315, suggesting a recent population expansion or selective sweep (Table 7).

AMOVA suggested a relatively high genetic diversity, with an 85.41% diversity within the populations, while there was only a 14.58% genetic diversity among the populations.

The Fst test value was relatively low, indicating that the populations were closely related with a value of 0.14585 (Table 8).

We also performed the Fst test among the *L. infantum* strains from different geographic areas. We excluded two regions (northwest and south) that were represented each by one strain. Overall, the Fst value demonstrated variant levels of genetic differentiation of *L. infantum* circulating in the included geographic areas (Table 9).

### 3.4. Genotypic Variation in L. infantum

From the 31 concatenated sequences, a total of 14 sequence types (STs) were deduced. The number of allele types at each locus were six at *gpi*, five at *alat*, *g6pd*, *me* and *pgd*, three at *pgm* and finally two at *fh*. This latter locus had the lowest discriminative power (0.065), while the locus *pgd* had the highest discriminative power with a value of 0.634 (Table 10).

### 3.5. Haplotype Diversity of L. infantum

The number of haplotypes generated for each locus ranged between three in *fh* as the least polymorphic locus and seven in the markers *gpi* and *pgd* as the most polymorphic ones. In all the seven markers, the most common haplotype had strains from at least four geographic areas, and most of them had strains from different forms: CL, VL and CanL. In every network, at least two haplotypes were singletons including one strain each (Table 1).

**Figure 2:**
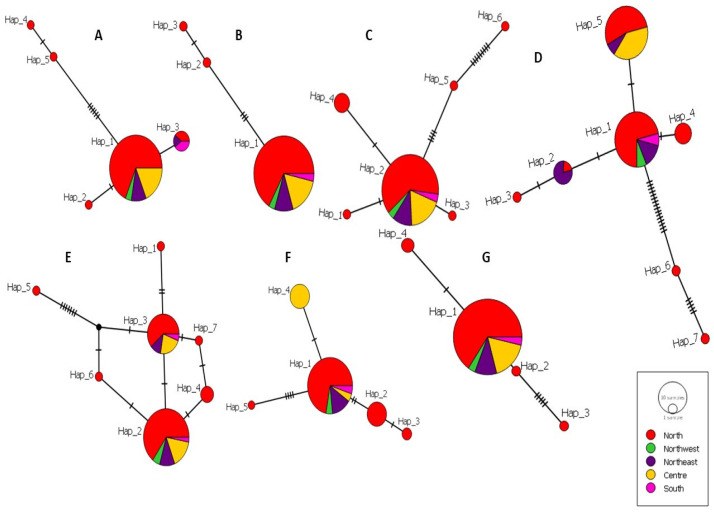
The haplotype networks of each gene: (**A**) *alat*; (**B**) *fh*; (**C**) *g6pd*; (**D**) *pgd*; (**E**) *gpi*; (**F**) *me*; (**G**) *pgm.* Circle size reflects the frequency of haplotypes and color represents the geographical origin. The black bars represent nucleotide changes.

We created the haplotype network of each gene separately; the seven haplotype networks had different patterns. The loci *fh* and *pgm* had the simplest networks with a straight-line pattern, and the loci *g6pd*, *pgd* and *gpi* were the most variable and displayed the most complex network patterns. The number of mutational events ranged from one mutation as the most frequent event and sixteen mutations that appeared once in *pgd* network (Figure 2).

### 3.6. Phylogenetic Analysis of L. infantum

The phylogenetic tree was constructed to display the evolutionary biology of *L. infantum* using the seven polymorphic loci. *L. major* Friedlin and *L. tropica* CDC216-162 strains were used as outgroups.

**Figure 3:**
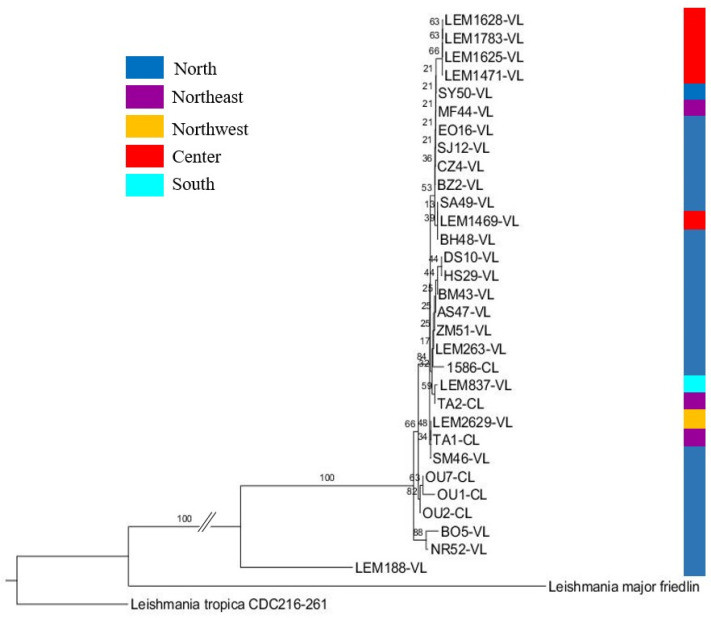
Phylogenetic tree of *L. infantum* based on the concatenated sequences of the seven loci used for the MLST analysis with the disease form indicated.

All the *L. infantum* strains clustered together except for one strain, LEM188, which showed a prominent differentiation from the rest of the strains consistently with the haplotype network, where it was represented by Hap_14 and Hap_15, which was far from the closest haplotype Hap_13 (Figure 3).

The samples from the same geographic area clustered together (north and center), suggesting a correlation between the geographic area and the clustering. Three strains from the northern macrofocus (the northeast, the north and the northwest) clustered together (Figure 3).

Based on the disease form, almost all of the VL strains and canine strains clustered together into different groups; half of the CL strains formed a group on their own, while the other CL strains clustered with VL and CanL strains in different groups. However, the CL/HIV+ strain differentiated on its own (Figure 3).

### 3.7. Recombination among L. infantum Strains

The estimate of the recombination parameter (R) per gene was 0.001, and the minimum number of recombination events (Rm) recorded four events; these recombination events were detected between the following sites (11, 1045)-(1393, 1492)-(1492, 1783) and (2028, 2575).

**Figure 4:**
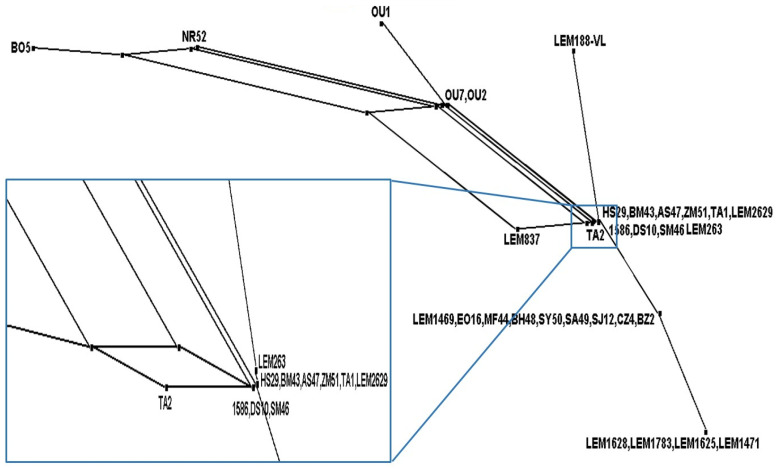
Splits decomposition network of the concatenated DNA sequences of the seven loci for thirty-one *L. infantum* strains based on the neighbor net method.

The splits network based on the neighbor net method using the alignment of the concatenated sequences for seven loci of all the *L. infantum* strains suggested through the presence of parallelograms and the network-like structure the occurrence of recombination among the *L. infantum* strains (Figure 4).

### 3.8. Temporal Variation Analysis

**Figure 5:**
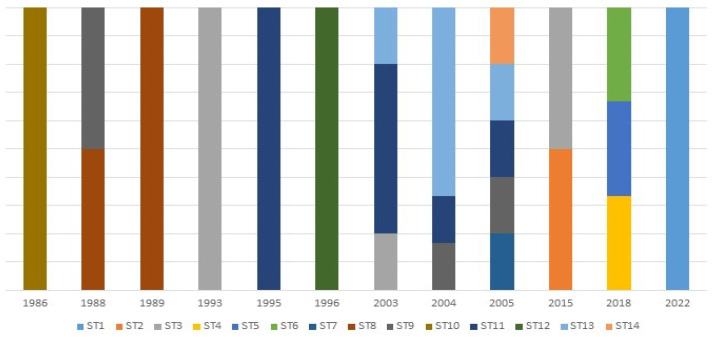
Temporal variation in sequence types (STs) based on the seven markers MLST scheme for *L. infantum*.

The temporal variation in the different sequence types for all the *L. infantum* strains was assessed. An association between the STs and the year of isolation was observed; out of the fourteen detected sequence types, nine were unique as they appeared only once; ST1 in 2022, ST2 in 2015, ST4 in 2018, ST5 in 2018, ST6 in 2018, ST7 in 2005, ST10 in 1986, ST12 in 1996 and ST14 in 2005. The other sequence types were relatively constant over the years; ST13 included strains from 2003–2005, ST11 included strains isolated between 1995 and 2003–2005, ST9 included strains from 1988, 2004–2005, ST8 appeared in 1988 and 1989 and ST3 included strains from 1993, 2003 and 2015 (Figure 5).

### 3.9. Analysis of the Genetic Exchange between L. infantum and L. tropica

The possibility of gene flow between the two endemic *Leishmania* species *L. infantum* and *L. tropica* in Morocco in two sympatric foci in the north and center was assessed. An additional set of the same seven markers for the fourteen *L. tropica* strains were used, twelve from the center and two from the north. The sequences of five markers (*me*, *pgd*, *fh*, *g6pd* and *gpi*) for the twelve strains of *L. tropica* were produced in our laboratory and already deposited in GenBank (Table 3), and the two missing markers, *pgm* and *alat*, were amplified and sequenced for these twelve *L. tropica* strains. In addition, the seven markers were amplified for the two other *L. tropica* strains from the north, whose sequences were submitted to GenBank and are accessible using the following accession numbers: OQ627481 to OQ627482 (for *alat*), OQ627545 to OQ627546 (for *fh*), OQ627578 to OQ627579 (for *g6pd*), OQ627611 to OQ627612 (for *gpi*), OQ627644 to OQ627645 (for *me*), OQ627677 to OQ627678 (for *pgd*) and OQ627710 to OQ627711 (for *pgm*).

#### 3.9.1. Genotypic Variation Analysis of *L. infantum* and *L. tropica*

Twenty-two sequence types (STs) were deduced from forty-five concatenated sequences of *L. infantum* and *L. tropica*. The highest number of allele types was nine at *fh* and *pgd* and the lowest was at *alat*. The discrimination power and the typing efficiency were calculated for each locus (Table 11).

#### 3.9.2. Haplotypes Diversity of *L. infantum* and *L. tropica*

The number of haplotypes for each marker ranged between six for the marker alat and eleven for the marker pgd. None of the haplotypes in all the markers had *L. infantum* and *L. tropica* strains together. Some haplotypes were private as they included strains of *L. tropica* from the same focal population, and others had *L. tropica* strains from two different population, namely the center and the north. Singleton haplotypes were present in all the markers for having one *L. tropica* strain from the north, the center or both (Table 1).

#### 3.9.3. Phylogenetic Analysis of *L. infantum* and *L. tropica*

The phylogenetic tree was constructed to display the evolutionary biology of *L. infantum* and *L. tropica* using the seven polymorphic loci. *L. major* Friedlin was used as an out group.

**Figure 6:**
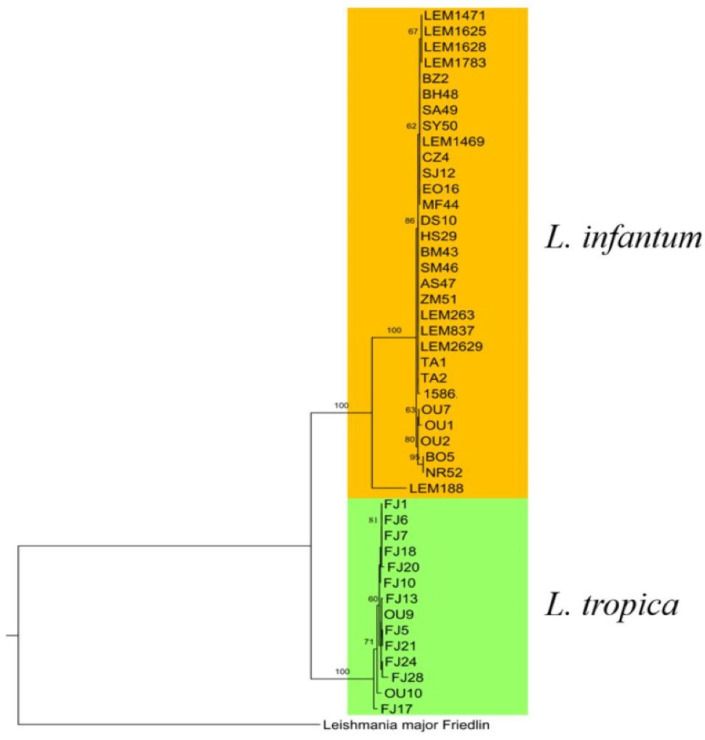
Phylogenetic tree of the concatenated sequences of *L. infantum* and *L. tropica* of the seven loci used for the MLST analysis.

In the phylogenetic tree, each species formed a cluster on its own. Inside *L. tropica*’s cluster, all of the center samples formed a group including (OU9) from the north, with the exception of two samples, one from the center (FJ17) and one from the north (OU10), as each one of them clustered on its own. *L. tropica*’s strains (OU9 and OU10) were isolated from the same focus as *L. infantum*’s strains (OU1, OU2 and OU7), and all the (FJ) strains were also isolated from the same focus as *L. infantum*’s strains (LEM1469, LEM1471, LEM1625, LEM1628 and LEM1783). However, these strains clustered with their respective species (Figure 6).

## 4. Discussion

Among the typing techniques of *Leishmania* species and strains, multilocus enzyme electrophoresis (MLEE) is still considered to be the gold standard [20]. However, it has many drawbacks; it is slow, laborious, costly, bulk cultures of live parasites are necessary and it has a low discriminatory power due to its inability to detect nucleotide substitutions that do not change the amino acid composition [21]. For *Leishmania infantum*, the discriminatory power of MLEE for studying genetic variability among strains is limited because most *L. infantum* strains in the Mediterranean countries belong to the zymodeme MON-1 regardless of the disease form, host and immune status [41]; it represents approximately 70% of all identified strains [42].

In Morocco, the *L. infantum* zymodeme MON-1 is responsible for the visceral form, which is lethal if left untreated in over 95% of cases [43], while zymodeme MON-24 is responsible for the cutaneous form that has been increasing in terms of its geographic spread and number of cases [7]. Few molecular studies have been conducted on the population genetics of this clinically important species; thus, a better understanding of the dynamics of leishmaniasis due to *L. infantum* and the population structure of this species in Morocco through genetic techniques with high discriminatory power is needed.

Housekeeping genes with high and variable rates of sequence diversity within and between species or genera are the ideal targets for characterization of microorganisms including *Leishmania* [24,44,45,46]. The MLST approach is based on the sequence of a set of seven to ten housekeeping genes that result in each strain having a distinct numerical allelic profile, which is abbreviated to a unique identifier, i.e., the sequence type (ST) [47]. This approach has proved its usefulness in being an accurate and highly discriminative portable typing system [47]. This method is considered as a potentially powerful phylogenetic approach that is poised to become the new reference method for *Leishmania* species characterization in the near future [23].

In our study, the 31 concatenated *L. infantum* sequences showed a high level of heterogeneity with 82 polymorphic/heterozygous sites (S), whereas the PCR-RFLP-KDNA polymorphisms of 188 *L. infantum* strains from Morocco and Portugal were less significant with values of S = 19 and S = 30, respectively [41]. The same technique applied to 44 *L. infantum* sequences from southeastern Spain revealed a value of S = 11 [48].

Fourteen sequence types were deduced, showing the high intraspecific diversity of *L. infantum*. In Morocco, other studies and through different techniques have proven the diversity of this species; using the multilocus microsatellite typing (MLMT) method on thirty-three Moroccan strains showed thirty-three genotypes [49], and kDNA-PCR-RFLP on seventy-five samples generated six genotypes [41]. The PCR-RFLP analysis of a single-copy gene encoding N-acetylglucosamine-1-phosphate transferase (*nagt*) was employed on a population of forty Moroccan *L. infantum* strains showed the genetic heterogeneity with six different genotypes [25]. In Spain, four RFLP-kDNA genotypes were deduced in a population of forty-four *L. infantum* sequences [48]. In another study in the south of France, the MLMT method was employed to assess 270 *L. infantum* isolates using 12 microsatellites, and the results revealed a total of 121 different genotypes [50].

The MLST scheme used in our study revealed a significant level of heterogeneity in *L. infantum* in Morocco. Moreover, it showed a higher number of heterozygous sites and genotypes, confirming the high resolution and discriminatory power of this technique. This conclusion is supported by the results of Banu et al., (2019), where seven *L. infantum* isolates, four from the Old World and three from the New World, clustered into seven sequence types using MLST. In the same work, it was reported that MLST possesses sufficient discriminatory power to detect genetic heterogeneity within and between species of the *L. donovani* complex [51]. Moreover, the MLST scheme we used in this work had a discrimination power of 0.9, and, according to Hunter and Gaston (1988), a discrimination power of 0.9 implies that the results can be interpreted with confidence [52].

According to Lauthier et al. (2020), phylogeny based on each marker separately might be associated with some inconsistency among different genes. Therefore, concatenation is a powerful approach as it is based on a complete set of genes, as previously shown in *Leishmania* parasites [23]. In the phylogenetic tree constructed based on the concatenated sequences from the seven genes, most of the strains from the same geographic areas clustered together, which was in agreement with other studies that demonstrated a geographical clustering of *L. infantum* strains isolated in different regions [53,54].

For the two canine strains, the one from the center clustered with two VL strains from the north and the one from the south clustered with a strain from the northeast. Ferroglio et al. (2006), through a study conducted in northern Italy using kDNA, showed that certain RFLP patterns were associated with specific geographic regions while others were more widely distributed [55]. In addition, Alam et al. (2014), reported that two Chinese populations did not correlate exactly with their geographical origin clusters [56].

The phylogenetic tree showed clusters including human or canine strains only and groups with both sources. Moreover, most of the canine strains clustered with human (CL and VL) strains, which was in accordance with previous works in the country that proved the zoonotic character of the transmission cycle of *L. infantum* [15,18,49]. Other studies have also reported the clustering of human and canine isolates together [48,57].

According to Seridi et al. (2008), there is no strong confirmation of an association be-tween *L. infantum* clinical forms and geography in Algeria [58], partly because the in-dividual genetic susceptibility and host immune response influence the tropism for the skin or for internal organs [59,60]. However, in the phylogenetic tree, almost all of the VL human strains coming from the same geographic region (north) clustered together into different groups, and half of the CL strains from the north clustered together. This result was in agreement with the findings of Chargui et al. (2009), who found that two MON-24 populations could be differentiated in Tunisia [54]. One included strains isolated from CL mainly from the north, and the other included VL strains from the center of Tunisia. Nevertheless, due to the small sample size, this should be further studied to confirm whether it is a general trait or only caused by sampling bias [54].

Two CL strains from the northeast clustered with VL strains in two groups. This could be due to the genetic similarity of parasites causing both forms. Indeed, the two CL strains came from Taza, which has been known to be a hypoendemic focus of VL due to *L. infantum* since 1995 [61]. Alam et al. (2014) reported the genetic similarity between the strains causing VL or CL [56]. Kuhls et al. (2011) also reported that CL strains from Honduras formed a single cluster with one VL strain from the same origin [62]. Wang et al. (2010) reported that a strain isolated from a VL patient grouped with isolates from CL patients from a different province [63]. Noyes et al. (1997) and Belli et al. (1999) confirmed that the two clinical forms in Honduras and Nicaragua were caused by genetically similar parasites [64,65]. Moreover, the tropism of *L. infantum* zymodemes is not clear cut; they are known to cause both VL and CL, and the reasons leading to their respective clinical pictures seem to be very complex [62].

The CL/HIV+ strain had a unique sequence type (ST1), and it clustered on its own in the phylogenetic tree, which was in accordance with Cruz et al. (2002), who reported, in the case of *L. infantum*, that most of the newly discovered zymodemes were heterozygous [66]. In addition, the results of Alvar et al. (1997) reported that the enzymatic characterization of *Leishmania* isolates from HIV-positive patients from the Mediterranean basin showed an extreme variability in *L. infantum* in such patients; a total of 17 zymodemes were described in 150 *Leishmania* isolates [67]. Eight of the seventeen zymodemes, MON-136, MON-183, MON-185, MON-188, MON-190, MON-198, MON-199 and MON-201, were new zymodemes that have been encountered only in HIV-positive individuals [67]. Moreover, the zymodeme MON-183, previously described in two cases of VL/HIV-positive individuals [68], was confirmed as a new zymodeme responsible not only for VL but also for CL in coinfected patients [69].

Three of the northern CL strains clustered on their own in both the phylogenetic tree and the haplotype network indicating their homogeneity. This result could be explained by the recent emergence of CL due to *L. infantum* in Morocco, as the first case was reported in 1996, while VL was first notified in 1920 [7]. Momen et al. (1993) reported that a low diversity supports the hypothesis of a recent import of selected strains of *L. infantum* from the Old World to the New World [70]. This is in accordance with Marlow et al. (2014), who showed that the high homogeneity observed among *L. braziliensis* in the state of Santa Catarina, Brazil, is due to the recent emergence of this species in the latter state [71].

The human *L. infantum* strains showed less diversity and more similarity than the ca-nine strains. The geographic origin of the strains could explain this difference in diversity, as all the human strains belonged to the northern focus, i.e., the same ecosystem, while the canine strains came from different geographic areas (north, northwest, center and south) and therefore different ecosystems. These results were consistent with Ghatee et al. (2018), as they reported that the similarity of human sequence haplotypes of *L. infantum* from central areas in Iran is due, among other reasons, to the similarity of the ecosystems in these foci [72].

According to Mauricio et al. (2006), heterozygosity at several sites within a gene and at several loci can be a consequence of genetic exchange; mutation is a less likely explanation [26]. Many authors have reported that heterozygosity might suggest sexual re-production in the natural *Leishmania* population, even if asexual reproduction via clonal propagation is considered the main reproductive mechanism in the *Leishmania* genus [73,74,75]. Our results showed evidence of recombination between *L. infantum* strains through the presence of the network-like structure and the four recorded recombination events, which was consistent with Rogers et al. (2014), who reported through the analysis of 11 *L. infantum* entire genomes isolated from the vector evidence of a cross between two diverse strains with subsequent recombination [76]. Other studies have also reported the presence of recombination amongst *L. infantum* isolates [51,54,58].

In recent years, many *L. infantum* CL cases were reported in different parts of the country, mostly in endemic foci of *L. tropica*, indicating the invasion of *L. infantum* CL to the former species distribution areas [7]. In the phylogenetic tree, the *L. infantum* and *L. tropica* strains formed two clearly divergent clusters. In *L. tropica*’s cluster, one of the two northern strains clustered with the strains from the center, which could be due to infected human behavior, as they are considered to be the main reservoir of *L. tropica*; it could have been spread through the movement of human populations [77]. In the phylogenetic tree, there was no gene flow between *L. infantum* and *L. tropica* from the two studied foci. This could be explained by the fact that the appearance of *L. infantum* in *L. tropica*’s focus in central Morocco is a recent event, as this focus is the oldest *L. tropica* focus (Azilal province) [78]. The same goes for the northern focus (Ouazzane province), which is known to be endemic for VL, while CL appeared until 1997 [79].

Through the temporal variation analysis using the sequence types, an association between the STs and the year of isolation was observed, as most of the STs were unique. Conversely, Herrera et al. (2017) observed no association between the STs and the year of isolation in *L. panamensis* and *L. braziliensis* [80], and Gouzelou et al. (2013) also reported that no correlation to the year of strain isolation was identified [81]. On the other hand, Pomares et al. reported that some genotypes were detected during a limited period and are no longer detected, while other genotypes were still detected [50].

## 5. Conclusions

To the best of our knowledge, this study is the first to apply the MLST approach on *L. infantum* in Morocco. This technique revealed the high genetic diversity of this species and showed its zoonotic character. Moreover, this approach disclosed the existence of recombination between *L. infantum* strains. The co-existence of *L. infantum* and *L. tropica* in the two endemic foci is a recent event; therefore, no gene flow was detected. In future work, increasing the number of samples from different geographic areas and different sources, especially from the vector throughout the country and in foci where different species are sympatric, could be of great help to understand the dynamics of the species circulating in the country.

## Figures and Tables

**Table 1:** Geographical origin, clinical form, strain codes, sequence types and haplotypes of the studied samples of both species.

**Leishmania** **Species**	**Disease Form**	**Strain Code**	**Geographic Origin**	**Sequence Types**	**Haplotypes**						
					*alat*	*fh*	*g6pd*	*gpi*	*me*	*pgd*	*pgm*
*L. infantum*	HIV+/CL	1586	North	ST1	1–2	1	1–2	1–2	1	1	1
	CL	TA2	Northeast	ST2	1–3	1	2	2	1	1	1
	CL	TA1	Northeast	ST3	1	1	2	2–3	1	1	1
	CL	OU1	North	ST4	1	1	2	2	2–3	1	1
	CL	OU2	North	ST5	1	1	2	2	2	1	1
	CL	OU7	North	ST6	1	1	2–3	2	2–3	2–3	1
	VL	NR52	North	ST7	1	1	4	4	2	4	1
	Canine	LEM2629	Northwest	ST3	1	1	2	2	1	1	1
	Canine	LEM1783	Center	ST8	1	1	2	2–3	4	5	1
	Canine	LEM1628	Center	ST8	1	1	2	2–3	4	5	1
	Canine	LEM1625	Center	ST8	1	1	2	2–3	4	5	1
	Canine	LEM1471	Center	ST8	1	1	2	2–3	4	5	1
	Canine	LEM1469	Center	ST9	1	1	2	2	1	5	1
	Canine	LEM837	South	ST10	3	1	2	2–3	1	1	1
	Canine	LEM263	North	ST11	1	1	2	2–3	1	1	1
	Canine	LEM188	North	ST12	4–5	2–3	5–6	5–6	1–5	6–7	2–3
	VL	ZM51	North	ST11	1	1	2	2–3	1	1	1
	VL	SY50	North	ST13	1	1	2	2–3	1	5	1
	VL	SA49	North	ST9	1	1	2	2	1	5	1
	VL	BH48	North	ST9	1	1	2	2	1	5	1
	VL	AS47	North	ST11	1	1	2	2–3	1	1	1
	VL	SM46	North	ST3	1	1	2	2	1	1	1
	VL	MF44	Northeast	ST13	1	1	2	2–3	1	5	1
	VL	BM43	North	ST11	1	1	2	2–3	1	1	1
	VL	HS29	North	ST11	1	1	2	2–3	1	1	1
	VL	EO16	North	ST13	1	1	2	2–3	1	5	1
	VL	SJ12	North	ST13	1	1	2	2–3	1	5	1
	VL	DS10	North	ST11	1	1	2	2–3	1	1	1
	VL	BO5	North	ST14	3	1	4	4–7	2	4	4
	VL	CZ4	North	ST13	1	1	2	2–3	1	5	1
	VL	BZ2	North	ST13	1	1	2	2–3	1	5	1
*L. tropica*	CL	OU9	North	ST10	5	4–5	6	6	6	8	5
	CL	OU10	North	ST11	5	6	7–8	6	6	8	6
	CL	FJ1	Center	16	5	4	6	6	6	8–9	5
	CL	FJ5	Center	14	5	4–8	6	6	6	8	5
	CL	FJ6	Center	16	5	4	6	6	6	8–9	5
	CL	FJ7	Center	16	5	4	6	6	6	8–9	5
	CL	FJ10	Center	18	5–6	4–7	9–6	6	6	8	5
	CL	FJ13	Center	19	5	4	6	6	6	9–11	5
	CL	FJ17	Center	17	5	4	6	6–7	6–7	8–10	9
	CL	FJ18	Center	16	5	4	6	6	6	8–9	5
	CL	FJ20	Center	15	5	4–9	6	6	6	8–9	5
	CL	FJ21	Center	14	5	4–8	6	6	6	8–9	5
	CL	FJ24	Center	13	5	4	6	6	6	8	6
	CL	FJ28	Center	12	5	5–7	6	6	6	8	7–8

VL: visceral leismaniasis, CL: cutaneous leishmaniasis.

**Table 2:** Primer sequences and expected size of the amplified DNA markers used for the MLST scheme.

DNA Marker	Chromosome	Primers Sequences (5′ to 3′)	Amplicon Size (bp)
***alat***	12	FW: GTGTGCATCAACCCMGGGAA RE: CGTTCAGCTCCTCGTTCCGC	589
***fh***	29	FW: GTCATCGACGAYRATGAACG RE: CAACAAGARCGGCATYTACA	604
***gpi***	12	FW: TCCAAGTCRCAYATCAACGA RE: GCATTCGTCAACGTTTCTTG	500
***g6pd***	34	FW: GATYCGMGAGAAGGAGAATG RE: CGGTCGTTGTTGATGTTGAG	684
***me***	24	FW: AYCAGGTGGARCGGTACTGG RE: TGTGGAARCCAGCAGCKATG	687
***pgd***	35	FW: TGTGAGCHTGGCRAGAATCT RE: GTATCACAACGCTGGGGAGT	697
***Pgm***	21	FW: CAGAGAAGCTGACGTCCCG RE: GACGGGTTCACGAAGAAGG	529
***Cytb***	Kinetoplast Maxicircle	FW: AGCGGAGAGRARAGAAAG RE: GYTCRCAATAAAATGCAAC	618

**Table 3:** *Leishmania tropica* sequences retrieved from GenBank.

Marker	Accession Numbers
***fh***	MW411684.1,MW411686.1-MW411692.1,MW411694.1-MW411696.1,MW411703.1
***gpi***	MW411726.1,MW411728.1-MW411734.1,MW411736.1-MW411738.1
***g6pd***	MW411705.1,MW411707.1-MW411713.1,MW411715.1-MW411717.1,MW411723.1
***me***	MW411746.1,MW411748.1-MW411754.1,MW411756.1-MW411758.1,MW411765.1
***pgd***	MW411768.1,MW411770.1-MW411776.1,MW411778.1-MW411780.1MW411787.1

**Table 4:** The genetic diversity indices of all *L. infantum* concatenated strains.

Marker	Fragment Size	S	H	Hd	Pi	K	dN/dS
***alat***	412	9	5	0.23797	0.00145	0.596	0.5
***fh***	483	4	3	0.06399	0.00046	0.223	1
***g6pd***	594	16	6	0.24008	0.00123	0.731	0.333
***gpi***	447	12	7	0.56531	0.00208	0.932	0.333
***me***	485	8	5	0.49233	0.002	0.97	0.333
***pgd***	534	25	7	0.62507	0.00354	1.89	0.4705
***pgm***	431	8	4	0.12533	0.00082	0.352	0.1428
**Concatenated**	3368	82	18	0.89212	0.00168	5.694	-

S: number of polymorphic/heterozygous sites; H: number of haplotypes; Hd: haplotype diversity; Pi: nucleotide diversity; K: average number of nucleotide differences; dN/dS: the ratio of synonymous and non-synonymous substitutions.

**Table 5:** The genetic diversity indices of the canine and human groups.

Marker		*alat*	*fh*	*g6pd*	*gpi*	*me*	*pgd*	*pgm*	Concatenated
**Canine strains**	S	8	4	13	9	5	22	7	68
	H	4	3	3	4	3	4	3	9
	Hd	0.398	0.216	0.215	0.608	0.582	0.608	0.216	0.895
	Pi	0.00382	0.00153	0.00309	0.00333	0.00199	0.00829	0.00226	0.00355
	K	1.575	0.738	1.836	1.49	0.967	4.425	0.974	12.006
**Human strains**	S	2	0	1	4	3	4	1	17
	H	3	1	2	5	3	5	2	13
	Hd	0.17230	0	0.16913	0.5563	0.37632	0.62262	0.08879	0.86364
	Pi	0.00043	0	0.00035	0.00157	0.00167	0.00145	0.00021	0.00083
	K	0.17548	0	0.16913	0.70402	0.80761	0.77696	0.08879	2.8129

S: number of polymorphic/heterozygous sites; H: number of haplotypes; Hd: haplotype diversity; Pi: nucleotide diversity; K: average number of nucleotide differences.

**Table 6:** The genetic diversity indices of the human VL and CL sub-groups.

Marker		*alat*	*fh*	*g6pd*	*gpi*	*me*	*pgd*	*pgm*	Concatenated
**VL human strains**	S	1	0	1	2	2	2	1	9
	H	2	1	2	4	2	3	2	7
	Hd	0.121	0	0.226	0.608	0.299	0.613	0.121	0.814
	Pi	0.00029	0	0.00038	0.00159	0.00123	0.00139	0.00028	0.00070
	K	0.121	0	0.226	0.709	0.598	0.742	0.121	2.371
**CL Human strains**	S	2	0	2	3	3	2	0	12
	H	3	1	3	3	3	3	1	7
	Hd	0.318	0	0.318	0.318	0.666	0.318	0	0.864
	Pi	0.00081	0	0.00056	0.00142	0.00287	0.00088	0	0.00094
	K	0.333	0	0.333	0.636	1.394	0.469	0	3.167

S: number of polymorphic/heterozygous sites; H: number of haplotypes; Hd: haplotype diversity; Pi: nucleotide diversity; K: average number of nucleotide differences.

**Table 7:** Tajima’s D statistic of the different groups.

	**All Strains**	**Canine Strains**	**Human Strains**	
			−0.89377	
			CL strains	VL strains
**Tajima’s D statistic**	−2.31766 **	−1.63404	−0.85315	0.18667

**: *p*-value < 0.01.

**Table 8:** AMOVA among *L. infantum* from VL and CL forms and CanL and the differentiation test (Fst).

	Among (VL, CL and CanL)	
	Among Populations	Within Populations
**d.f.** *	2	59
**Sum of Squares**	15.012	104.472
**Variance Components**	0.30236	1.77072
**Percentage Variation**	14.58%	85.41%
**Fst**	0.14585	
***p*-value**	<0.0001	

* d.f.: degrees of freedom.

**Table 9:** Comparative Fst between *L. infantum* populations from different geographical origins.

	North	Northeast	Center
**North**	-	0.0117	0.226
**Northeast**	-	-	0.455

**Table 10:** Number of alleles, number of polymorphisms, typing efficiency and discrimination power.

Marker	Number of Alleles	Number of Polymorphisms	Typing Efficiency	Discrimination Power (95% CI) *
***alat***	5	9	0.556	0.299 (0.071–0.527)
***fh***	2	4	0.5	0.065 (0–0.194)
***g6pd***	5	16	0.312	0.299 (0.071–0.527)
***gpi***	6	12	0.5	0.594 (0.439–0.748)
***me***	5	8	0.625	0.527 (0.315–0.739)
***pgd***	5	25	0.2	0.634 (0.532–0.737)
***pgm***	3	8	0.375	0.127 (0–0.3)
**Total**	14	82	0.378	0.91 (0.858–0.962)

* 95% confidence interval.

**Table 11:** Number of alleles, number of polymorphisms, typing efficiency and discrimination power.

	Number of Alleles	Number of Polymorphisms	Typing Efficiency	Discrimination Power (95% CI) *
***alat***	5	24	0.208	0.511 (0.382–0.64)
***fh***	9	16	0.562	0.538 (0.364–0.712)
***g6pd***	8	29	0.276	0.598 (0.45–0.746)
***gpi***	6	18	0.333	0.566 (0.439–0.693)
***me***	7	16	0.438	0.699 (0.596–0.802)
***pgd***	9	41	0.22	0.797 (0.731–0.862)
***pgm***	7	18	0.389	0.543 (0.39–0.697)
**Total**	22	162	0.315	0.907 (0.849–0.966)

* 95% Confidence Interval.

## Data Availability

The reported sequences in the present paper were deposited in the GenBank database under the following accession numbers: OQ627483 to OQ627513 (for *alat*), OQ627514 to OQ627544 (for *fh*), OQ627547 to OQ627577 (for *g6pd*), OQ627580 to OQ627610 (for *gpi*), OQ627613 to OQ627643 (for *me*), OQ627646 to OQ627676 (for *pgd*), OQ627679 to OQ627709 (for *pgm*) for *L. infantum* and: OQ627481 to OQ627482 (for *alat*), OQ627545 to OQ627546 (for *fh*), OQ627578 to OQ627579 (for *g6pd*), OQ627611 to OQ627612 (for *gpi*), OQ627644 to OQ627645 (for *me*), OQ627677 to OQ627678 (for *pgd*) and OQ627710 to OQ627711 (for *pgm*) for *L. tropica*.

## Supplementary Materials

The supporting information can be downloaded at: https://www.mdpi.com/article/10.3390/pathogens12060785/s1. Figure S1. Agarose gel profiles of MLST genes: (A) *alat*: 589 bp; (B) *pgm*: 529 bp; (C) *me*: 687 bp; (D) *gpi*: 500 bp; (E) *pgd*: 697 bp; (F) *g6pd*: 684 bp; (G) *fh* 604 bp. NC: negative control; MW: 100 bp molecular weight marker.
