# Supplementary material for: Genetic Diversity and Population Structure of Leishmania infantum in Morocco as Revealed by Multilocus Sequence Typing (MLST) Approach
Source: Pathogens. 2023 May 31;12(6):785. doi: 10.3390/pathogens12060785 (PMC10304088; doi:10.3390/pathogens12060785)
Supplement: Supplementary file 1 [file pathogens-12-00785-s001.zip › pathogens-2380131-supplementary.pdf]

A

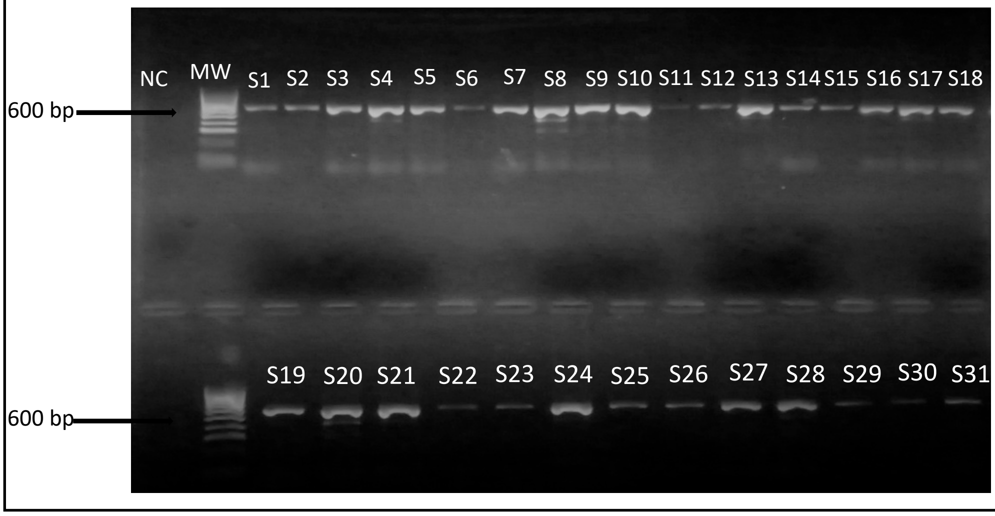
NC
MW
S1 S2 S3 S4 S5 S6 S7 S8 S9 S10 S11 S12 S13 S14 S15 S16 S17 S18
600 bp
S19 S20 S21 S22 S23 S24 S25 S26 S27 S28 S29 S30 S31
600 bp

B

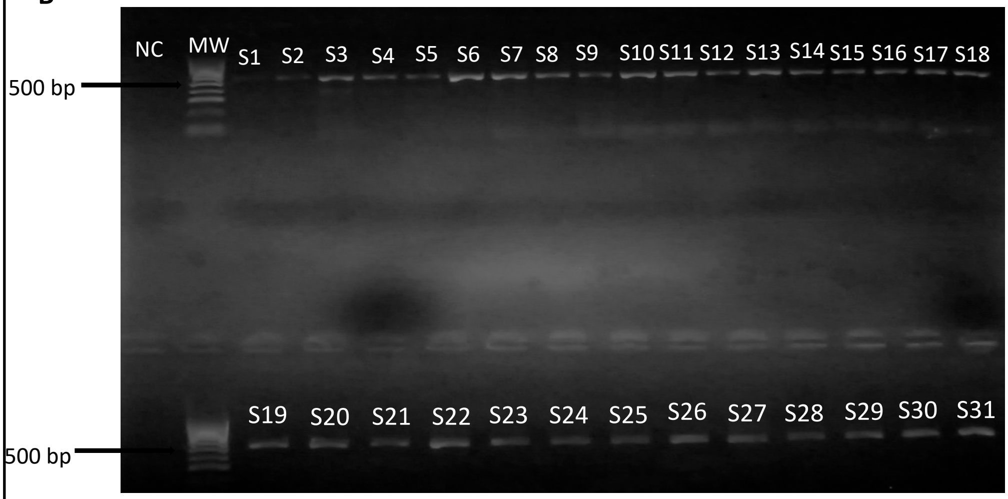
NC
MW
S1 S2 S3 S4 S5 S6 S7 S8 S9 S10 S11 S12 S13 S14 S15 S16 S17 S18
500 bp
S19 S20 S21 S22 S23 S24 S25 S26 S27 S28 S29 S30 S31
500 bp

C

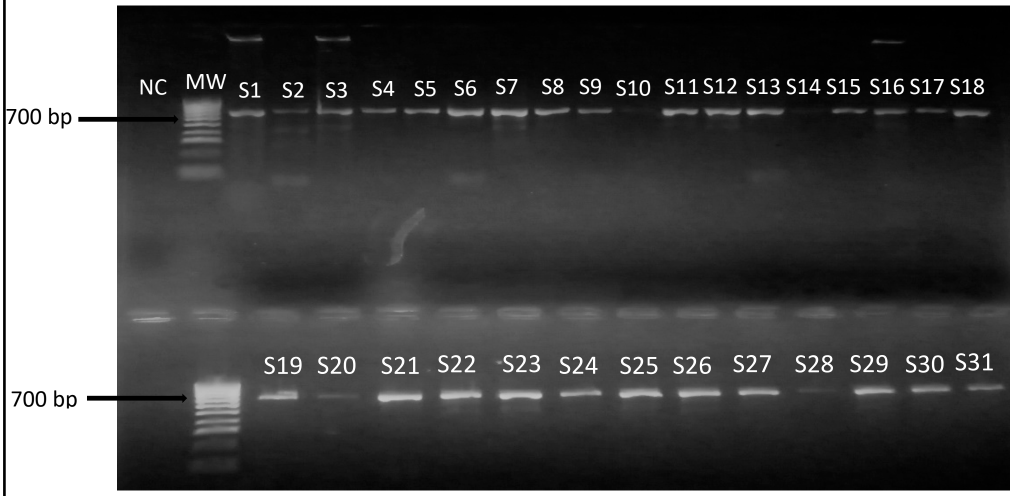
NC
MW
S1 S2 S3 S4 S5 S6 S7 S8 S9 S10 S11 S12 S13 S14 S15 S16 S17 S18
700 bp
S19 S20 S21 S22 S23 S24 S25 S26 S27 S28 S29 S30 S31
700 bp

D

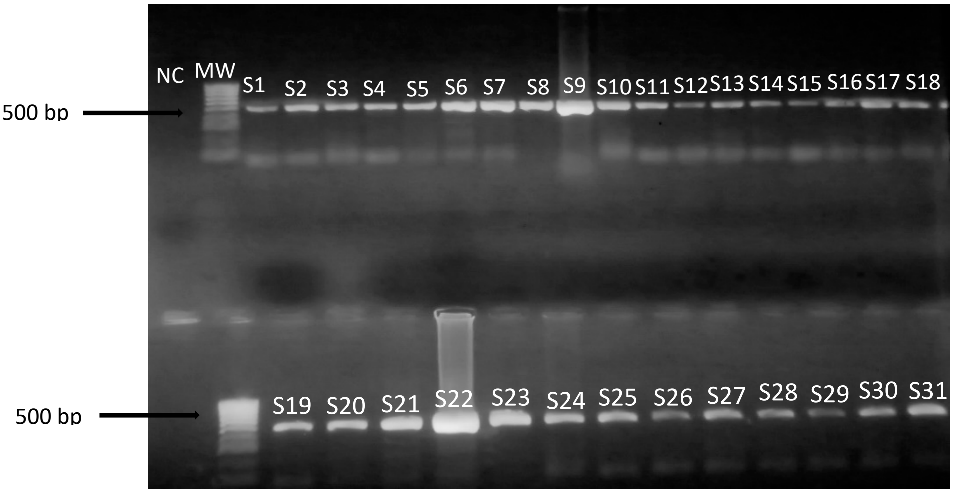
NC MW S1 S2 S3 S4 S5 S6 S7 S8 S9 S10 S11 S12 S13 S14 S15 S16 S17 S18
500 bp
S19 S20 S21 S22 S23 S24 S25 S26 S27 S28 S29 S30 S31
500 bp

E

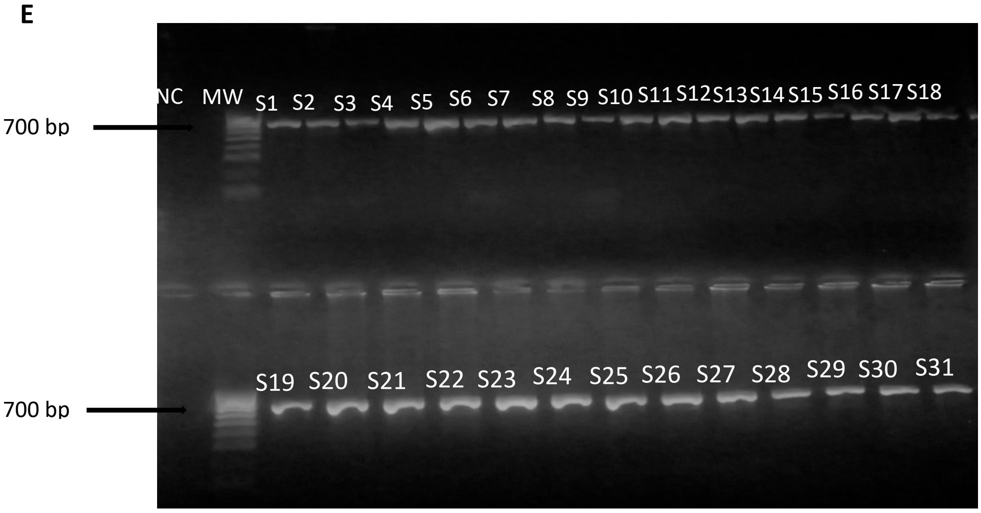
NC MW S1 S2 S3 S4 S5 S6 S7 S8 S9 S10 S11 S12 S13 S14 S15 S16 S17 S18
700 bp
S19 S20 S21 S22 S23 S24 S25 S26 S27 S28 S29 S30 S31
700 bp

F

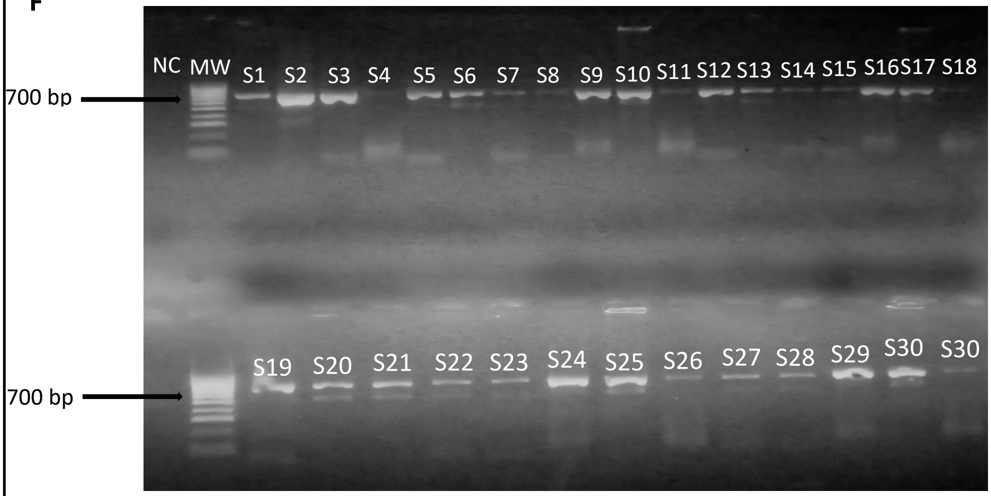
NC MW S1 S2 S3 S4 S5 S6 S7 S8 S9 S10 S11 S12 S13 S14 S15 S16 S17 S18
700 bp
S19 S20 S21 S22 S23 S24 S25 S26 S27 S28 S29 S30 S30
700 bp

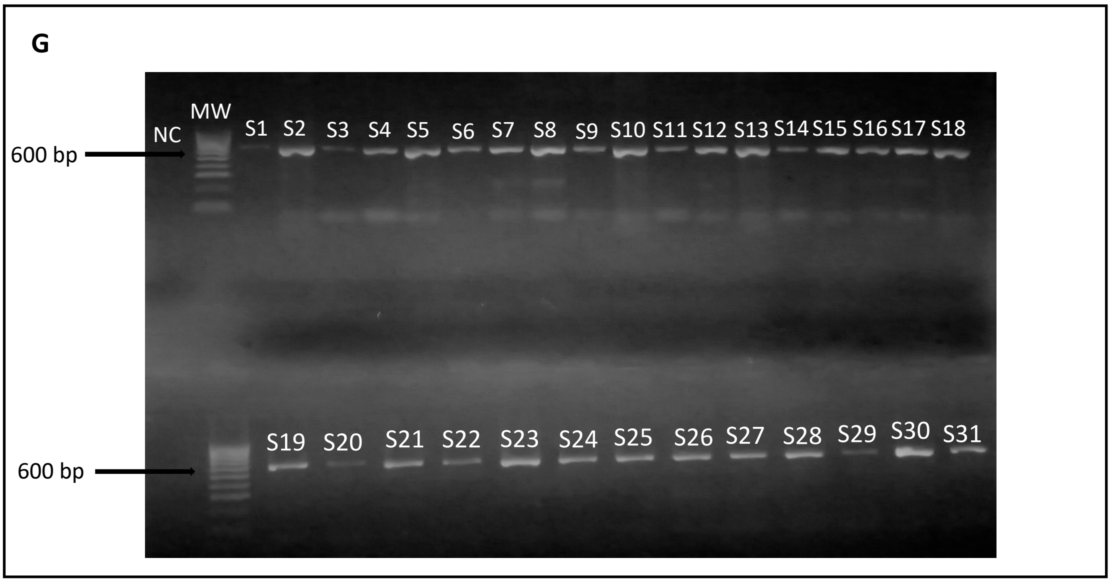

Figure S1. Agarose gel profiles of MLST genes: (**A**) *alat:* 589; (**B**) *pgm*: 529; (**C**) *me*: 687 bp; (**D**) *gpi:* 500 bp; (**E**) *pgd*: 697 bp; (**F**) *g6pd*: 684 bp; (**G**) *fh* 604 bp; and NC: negative control; MW: 100 bp molecular-weight marker.
